# Fine mapping and sequence analysis reveal a promising candidate gene encoding a novel NB-ARC domain derived from wild rice (*Oryza officinalis*) that confers bacterial blight resistance

**DOI:** 10.3389/fpls.2023.1173063

**Published:** 2023-08-25

**Authors:** Pragya Sinha, Dilip Kumar T., Hajira Sk, Manish Solanki, C. G. Gokulan, Ayyappa Das, Anila Miriyala, Rekha Gonuguntala, Punniakoti Elumalai, Kousik M. B. V. N, Masthani S. K., Chaitra Kumboju, Yugander Arra, Laha G. S., N. Neerja Chirravuri, Hitendra Kumar Patel, Irfan Ahmad Ghazi, Sung-Ryul Kim, Kshirod K. Jena, Surekha Rani Hanumanth, Ricardo Oliva, Satendra K. Mangrauthia, Raman Menakshi Sundaram

**Affiliations:** ^1^ Department of Biotechnology, ICAR-Indian Institute of Rice Research, Hyderabad, India; ^2^ Crop Improvement, Council of Scientific & Industrial Research (CSIR)-Centre for Cellular and Molecular Biology, Hyderabad, India; ^3^ Seeds/Biotech R&D Division, Rallis India Limited, Bangalore, India; ^4^ Institute of Molecular Physiology, Heinrich Heine University, Düsseldorf, Germany; ^5^ Crop Improvement, Academy of Scientific and Innovative Research (AcSIR), Ghaziabad, India; ^6^ Department of Plant Sciences, School of Life Sciences, University of Hyderabad, Hyderabad, India; ^7^ Rice Breeding Innovation Platform, International Rice Research Institute (IRRI), Los Banos, Philippines; ^8^ School of Biotechnology, Kalinga Institute of Industrial Technology (KIIT) University, Bhubaneswar, Odisha, India; ^9^ Department of Genetics, Osmania University, Hyderabad, India; ^10^ Safe and Sustainable Value Chain, World Vegetable Center, Tainan, Taiwan

**Keywords:** bacterial blight (BB), *Xanthomonas oryzae* pv. *oryzae*(*Xoo*), *Oryza officinalis*, recombinant inbred line (RIL), mapping, single nucleotide polymorphism (SNP), nucleotide-binding adaptor (NB-ARC)

## Abstract

Bacterial blight disease of rice caused by *Xanthomonas oryzae* pv. *oryzae* (*Xoo*) is one of the most serious constraints in rice production. The most sustainable strategy to combat the disease is the deployment of host plant resistance. Earlier, we identified an introgression line, IR 75084-15-3-B-B, derived from *Oryza officinalis* possessing broad-spectrum resistance against *Xoo*. In order to understand the inheritance of resistance in the *O. officinalis* accession and identify genomic region(s) associated with resistance, a recombinant inbred line (RIL) mapping population was developed from the cross Samba Mahsuri (susceptible to bacterial blight) × IR 75084-15-3-B-B (resistant to bacterial blight). The F_2_ population derived from the cross segregated in a phenotypic ratio of 3: 1 (resistant susceptible) implying that resistance in IR 75084-15-3-B-B is controlled by a single dominant gene/quantitative trait locus (QTL). In the F_7_ generation, a set of 47 homozygous resistant lines and 47 homozygous susceptible lines was used to study the association between phenotypic data obtained through screening with *Xoo* and genotypic data obtained through analysis of 7K rice single-nucleotide polymorphism (SNP) chip. Through composite interval mapping, a major locus was detected in the midst of two flanking SNP markers, viz., Chr11.27817978 and Chr11.27994133, on chromosome 11L with a logarithm of the odds (LOD) score of 10.21 and 35.93% of phenotypic variation, and the locus has been named *Xa48t*. *In silico* search in the genomic region between the two markers flanking *Xa48t* identified 10 putatively expressed genes located in the region of interest. The quantitative expression and DNA sequence analysis of these genes from contrasting parents identified the *Os11g0687900* encoding an NB-ARC domain-containing protein as the most promising gene associated with resistance. Interestingly, a 16-bp insertion was noticed in the untranslated region (UTR) of the gene in the resistant parent, IR 75084-15-3-B-B, which was absent in Samba Mahsuri. The association of *Os11g0687900* with resistance phenotype was further established by sequence-based DNA marker analysis in the RIL population. A co-segregating PCR-based INDEL marker, Marker_Xa48, has been developed for use in the marker-assisted breeding of *Xa48t*.

## 1 Introduction

Rice is one of the world’s main staple food crops. Sustainable rice production, which aims to reduce production losses due to pests and diseases, is an important element in ensuring global food security (FAO, 2004). Rice production is affected significantly by the number of diseases caused by bacteria, viruses, fungi, nematodes, etc.; bacterial blight (BB) caused by *Xanthomonas oryzae* pv. *oryzae* (*Xoo*) is one of the major constraints on higher productivity. The reduction in yield due to BB has recorded up to 81% in a severe case of infection (Zhang and Wang, 2013).

Host plant resistance through the breeding of one or more resistance genes is the most potent way of managing the yield losses due to the disease (Kumar et al., 2020). As the pathogen is highly dynamic in nature, it is equally important to extend the genetic repertoire from diverse novel sources including wild relatives of rice in response to the BB (Brar et al., 1997). Identification of genetic resources through the analysis of wild relatives and introgression of two or more major resistance genes with divergent functions into elite rice varieties are two promising approaches that can lead to durable resistance. Till now, 47 resistance genes have been mapped on various chromosomes for the BB, and many of the resistance genes have been identified and characterized from wild rice lines like *Xa21* (*Oryza longistaminata*), *Xa23* and *Xa30* (*Oryza rufipogon*), *Xa27* (*Oryza minuta*), *Xa29* (*Oryza officinalis*), *Xa33* and *Xa38* (*Oryza nivara*), and others (Song et al., 1995; Zhang et al., 2001; Gu et al., 2004; Tan et al., 2004; Natrajkumar et al., 2012). *O. officinalis*, one of the wild rice species, is the key resource for identifying numerous potential genes against many diseases due to their abundant genetic diversity. According to Zhang et al. (1994), *O. officinalis* makes up 50% of the highly resistant materials among the Chinese wild rice species. A recently published article by Matt Shenton et al. (2020) suggests that the genome size of *O. officinalis* is 1.6 times larger than the genome size of cultivated *Oryza sativa*, and genome analysis also revealed the prevalence of their stress tolerance to environmental biotic and abiotic changes. Expression pattern analysis of known BB resistance genes in *O. officinalis* indicated the possibility of the presence of new genes or quantitative trait loci (QTLs) in this wild species. There has been limited investigation on bacterial blight resistance in *O. officinalis*, and the molecular mechanism underlying the resistance has not been studied so far (Jiang et al., 2019). The wild rice introgression line derived from *O. officinalis* provides the solid foundation to identify the novel gene(s) or QTL against BB resistance.

In our study, keeping the above-mentioned information, we have conceptualized the experiment to identify the potential donor with broad-spectrum resistance and to study the pattern of inheritance of resistance. This study aims to map a novel resistance gene/QTL conferring resistance against BB disease in *O. officinalis* and identify putative candidate genes and associated markers through high-resolution genetic linkage mapping, *in silico* identification of putative candidate gene(s), and their validation through sequencing and expression analysis. The study holds immense significance, as it provides valuable insights into the genetic basis and molecular mechanisms associated with resistance against BB disease, as it can be leveraged for developing resistant cultivars through marker-assisted breeding.

## 2 Material and methods

### 2.1 Plant material and development of mapping population

The wild rice introgression line, IR 75084-15-3-B-B, containing introgressions from *O. officinalis*, displayed a high level of resistance against many virulent isolates of *Xoo*, viz., IX-020, IX-007, IX-212, and IX-206, collected from various parts of India. It was crossed with Samba Mahsuri, a high-yielding, fine grain-type rice variety, which is highly susceptible to BB disease. The true F_1_s derived from the cross were advanced by repeated selfing to develop a recombinant inbred line (RIL) population consisting of 400 individuals. In the F7 generation, a set of 94 lines consisting of 47 homozygous resistant and 47 homozygous susceptible was used for molecular mapping and identification of the genomic regions associated with the resistance to BB disease.

### 2.2 Screening of mapping population against *Xoo* strains

A virulent strain of *Xoo* isolate, i.e., IX-020 (collected from ICAR-IIRR, Hyderabad, Telangana, GPS coordinates 17.3201° N, 78.3939° E) [Yugander et al., 2017], was used for phenotypic evaluation of the mapping population developed from the above-mentioned cross. The isolate was cultured on modified Wakimoto’s agar (MWA) plates and incubated at 28°C for 72–96 hours (Laha et al., 2009). The bacterial cells were collected after the incubation period and diluted with sterile distilled water to a final concentration of approximately 10^8^ cfu/ml, which served as the inoculum. Under field conditions, the plants were inoculated at the maximum tillering stage through the leaf clipping method (Kauffman, 1973), and a minimum of five leaves per plant were clip inoculated.

The plants were scored for their reaction against *Xoo* following the Standard Evaluation System scale developed by International Rice Research Institute (IRRI-SES; http://www.knowledgebank.irri.org/images/docs/rice-standard-evaluation-system.pdf). Disease reaction was recorded 15 days post inoculation on the basis of average lesion length as well as disease score for each plant. The plants with a lesion length of less than 3 cm were classified as resistant (R; score 1), those with a lesion length of within 3–5 cm were classified as moderately resistant (MR; score 3), and those with a lesion length of more than 5 cm were classified as susceptible (S; score 5 or 7). The resistant parent IR 75084-15-3-B-B and susceptible parent Samba Mahsuri, their F_1_ progeny, and F_2_ individuals were screened and evaluated for the genetic inheritance of resistance. The disease scores for each F_2_ individual (approximately 900) were recorded to compute the segregation ratio using the chi-square test and for determining the number of genes involved in resistance at F_3_ generation; approximately 20 plants for each F_3_ line were raised, and all were screened for their disease reaction (minimum of five leaves per plant) using the isolate IX-020. Based on this observation, entries were considered as homozygous resistant if all the 20 plants showed resistance reaction, homozygous susceptible if susceptible reaction was recorded for all the 20 plants, and segregating if both resistant and susceptible reactions were observed within the plants. The homozygous lines were advanced through selfing to F_7_ generation. In the F_7_ generation, 94 homozygotes RILs were selected including 47 homozygous resistant and 47 homozygous susceptible lines for molecular mapping using single-nucleotide polymorphism (SNP) markers. In order to confirm the pattern of resistance observed in the resistant parent, a set of 40 lines consisting of 20 homozygous resistant lines and 20 homozygous susceptible lines along with the parents was screened against isolates, including IX-007, IX-212, and IX-206. The objective of this screening was to determine if the resistance pattern observed in the resistant parent was replicated in the selected lines.

### 2.3 SNP genotyping and construction of linkage map

Based on phenotypic screening for consecutive generations, 94 lines from the F_7_ generation consisting of 47 each of homozygous resistant and homozygous susceptible lines along with the parents Samba Mahsuri and IR 75084-15-3-B-B, were selected for molecular mapping. The selected lines were genotyped using a 7k rice SNP chip [platform Illumina iScan and SNP Chip details: Cornell-IR_RiceLD (Ref:15069608 X 339319)], which was composed of 6,658 SNPs (Morales et al., 2020). The polymorphic SNPs between the parents were identified from the software QTL IciMapping to construct the genetic linkage map through the inclusive composite interval mapping method (ICIM) using the Kosambi function in the software (https://www.isbreeding.net).

### 2.4 QTL mapping

For mapping the QTL associated with the BB resistance, genotypic data obtained from the 7k SNP chip were compared with the phenotypic data, i.e., BB resistance score as well as lesion length of the selected 94 entries. The analysis of QTL was executed using inclusive composite interval mapping for additive QTL (ICIM-ADD) using the software QTL IciMapping. As per the ICIM software, a logarithm of the odds (LOD) score of 3 was set as a minimum threshold based on the permutation test for the identification of QTLs with a significant effect on the phenotype.

### 2.5 *In silico* analysis for annotated putative candidate genes

The identified QTL region related to the BB resistance was analyzed using the Batch retrieval function from RAP-DB (https://rapdb.dna.affrc.go.jp/) as well as the Rice genome annotation project (http://rice.plantbiology.msu.edu/) for *in silico* analysis. The putative candidate genes were identified from the Rice annotation project Database website (https://rapdb.dna.affrc.go.jp) from the genomic region specific for the resistance QTL/gene.

### 2.6 RNA extraction and quantitative real-time PCR

To analyze the expression of the gene(s) present within the QTL interval as well as to identify the most likely putative candidate gene(s) for *Xa48t*, real-time PCR primers (**Supplementary Table 1**) were designed from the exonic regions of the genes using qPCR primer design tool QuantPrime (Arvidsson et al., 2008). Resistant parent IR 75084-15-3-B-B, susceptible parent Samba Mahsuri, and two homozygotes (one homozygous resistant and one homozygous susceptible) were grown on hydroponics media under controlled conditions with precise temperature, lighting, humidity, nutrient solution, water quality, etc., favoring *Xoo* spread on rice plant. Twenty-five-day- old seedlings were inoculated with *Xoo* isolate IX-020, and samples at 0 hpi (mock-inoculated plant) (hour post inoculation), 24 hpi, and 48 hpi were harvested for the analysis. Total RNA was extracted from each genotype in three replicates using the NucleoSpin^®^ RNA Plant kit and converted into cDNA using PrimeScript™ 1^st^ strand cDNA Synthesis Kit according to the protocol for the real-time PCR. TB Green^®^ Premix Ex Taq™ II along with diluted cDNA was analyzed under the qPCR system for gene expression analysis.

### 2.7 Marker development

To develop PCR-based diagnostic markers for *Xa48t*, we utilized the chromosome walking method (Chinault and Carbon, 1979). This method involves sequence analysis of the genes located within the region of interest, which were shortlisted based on the results of expression analysis through amplification using overlapping primers. To identify sequence differences between the resistant parent IR 75084-15-3-B-B and the susceptible parent Samba Mahsuri, overlapping primer pairs were designed for PCR amplification and sequencing. It was ensured that the expressed regions of the shortlisted genes, along with approximately 1-kb upstream and 500-bp downstream sequences of the putative candidate genes, were also sequenced. We also utilized the available simple sequence repeat (SSR) and INDEL markers within the region of interest on Chr. 11L. These markers were identified from the Rice Annotation Project Database (RAP-DB, http://rapdb.dna.affrc.go.jp/), and the synthesized SSR and INDEL markers were used for amplification of the parents to identify polymorphic markers. Detailed information regarding the markers is presented in **Supplementary Table 2**.

## 3 Results

### 3.1 BB resistance evaluation of mapping population

The resistant parent was observed to show a high level of resistance against the *Xoo* isolate with a lesion length of less than 1 cm, while the susceptible parent Samba Mahsuri recorded a susceptibility reaction with a lesion length of more than 9 cm. The F_1_s derived from the cross IR 75084-15-3-B-B/Samba Mahsuri were resistant with a lesion length of below 1 cm, indicating the dominant nature of BB resistance in IR 75084-15-3-B-B.

The F_2_ population was segregated in the ratio of 3:1, including 598 resistant and 229 susceptible lines. The observed frequency of the population segregation fitted the expected phenotypic ratio of 3:1, which was confirmed statistically through the chi- square test (**Table 1**).

**Table 1:** Frequency of resistance reaction against the *Xoo* isolate of F_2_ from the cross between IR 75084-15-3-B-B and Samba Mahsuri.

Population	No. of progeny	Total	Segregation ratio	p-V alue
F_2_	598 (resistant)	827	3:1	0.073
	229 (susceptible)			

Xoo, Xanthomonas oryzae pv. oryzae.

### 3.2 Identification of homozygous resistant and homozygous susceptible lines for QTL mapping

**Figure 1:**
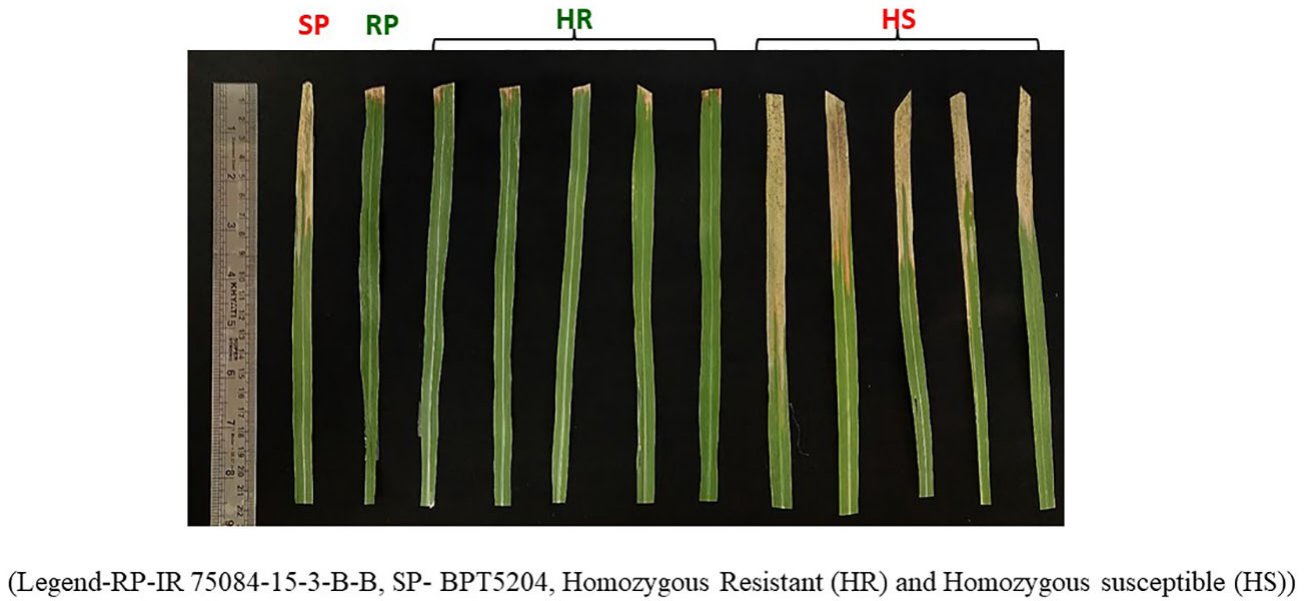
Bacterial blight disease reaction on identified homozygous resistant and identified homozygous susceptible lines along with the parents. RP-IR 75084-15-3-B-B, SP- BPT5204, homozygous resistant (HR), and homozygous susceptible (HS).

From F_2_s, a population consisting of 400F_3_ lines (300 resistant and 100 susceptible) was selected and screened for BB resistance under field conditions along with the parents. Among them, 160 F_3_ lines derived displayed resistance, while the 100 F_3_s were susceptible to the BB disease. The selected lines were advanced further through selfing to F_7_ generation, and finally, a set of 94 lines (47 homozygous resistant lines and 47 homozygous susceptible lines) were identified and used for QTL mapping. The selected 94 F_7_ lines (i.e., recombinant inbred lines) along with the parents were also screened under controlled glasshouse conditions in order to confirm their homozygosity and were then used for mapping (**Figure 1**). Again, the screening of 40 homozygous sets displayed a resistance pattern of 20 homozygous resistant lines similar to that of the resistant parent when exposed to *Xoo* isolates IX-007, IX-212, and IX-206.

However, the homozygous susceptible lines showed a consistent susceptibility pattern across the isolates tested (**Supplementary Table 4**).

### 3.3 Construction of linkage map

**Figure 2:**
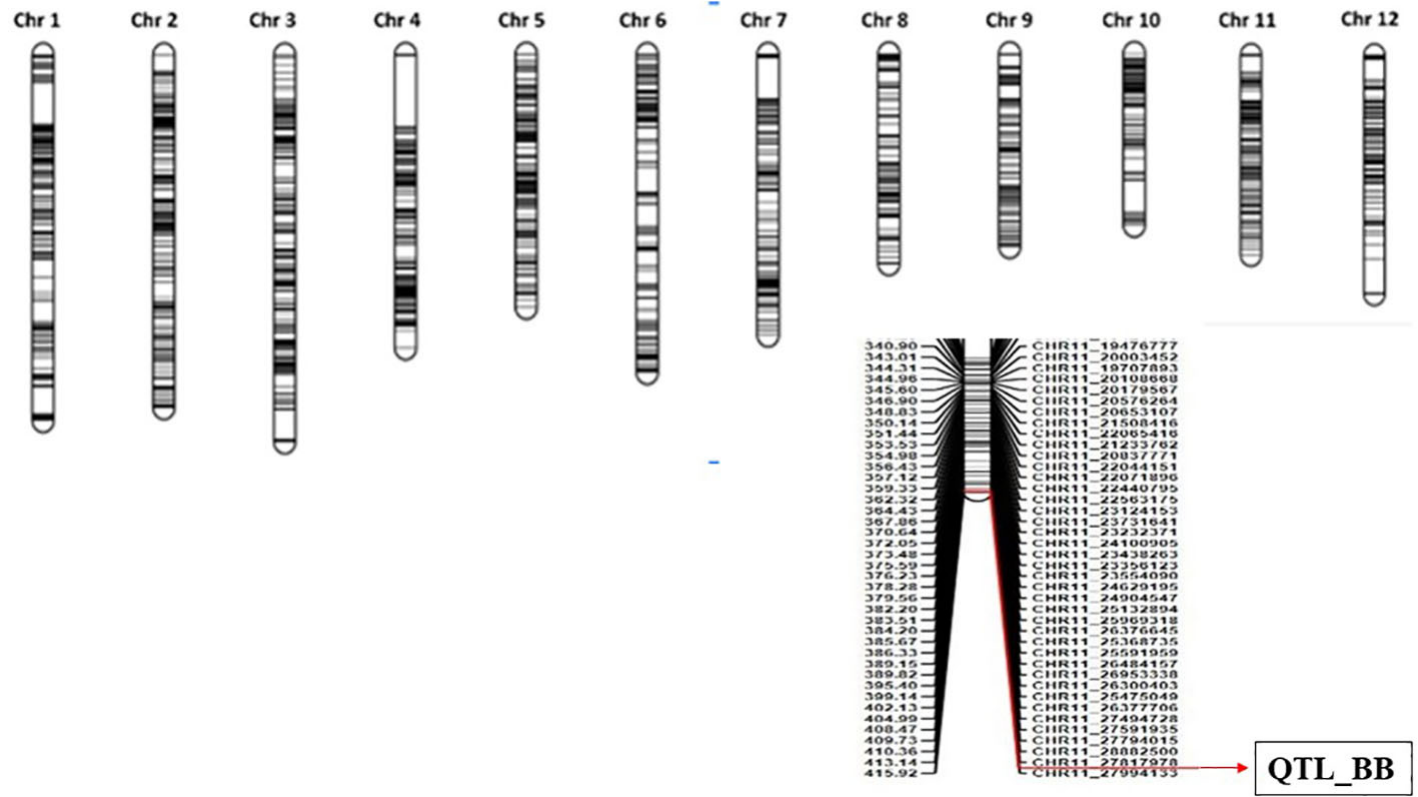
Genetic linkage map of the rice genome constructed based on the recombination frequencies of 1,014 SNP markers. The major QTL associated with bacterial blight resistance was located on chromosome 11. SNP, single-nucleotide polymorphism; QTL, quantitative trait locus.

A high-density genetic linkage map was constructed using IciMapping software (https://www.isbreeding.net) with the Kosambi function from the phenotyping data pertaining to BB scores of the mapping population and the genotype data obtained from 7k data analysis. An aggregate of 6,658 SNP markers were called for genotyping of 94 selected RILs and the parents. The SNP markers with insertion/deletion or either heterozygous condition in either of the parents were filtered out from the polymorphic survey. Based on shortlisting of the polymorphic survey of the SNP markers, which were in the homozygous state, we identified 2,562 SNPs for further linkage analysis. Out of these, 1,514 redundant SNPs were found, which overlapped the same position on each chromosome with 0 cM through linkage map information supported by the software. The linkage map was finally constructed with a total of 1,048 SNPs, with approximately 88 SNPs anchored in each chromosome on average (**Table 2**; **Figure 2**).

**Table 2:** Distribution of SNP markers on all the chromosomes used for construction of linkage map and QTL analysis.

Chromosome	Tested marker	Polymorphic markers	Selected marker	Map distance (cM)	Density (SNPs/cM)
Chromosome 1	850	270	109	281	0.38
Chromosome 2	660	274	123	250	0.49
Chromosome 3	683	319	117	288	0.40
Chromosome 4	646	225	94	200	0.47
Chromosome 5	509	210	92	177	0.51
Chromosome 6	551	203	89	188	0.47
Chromosome 7	524	147	83	198	0.41
Chromosome 8	545	170	60	151	0.39
Chromosome 9	455	185	67	148	0.45
Chromosome 10	472	169	55	122	0.45
Chromosome 11	631	223	86	219	0.39
Chromosome 12	572	167	73	180	0.40
**Total**	**7,098**	**2,562**	**1,048**	**2,402**	**5.21**

SNP, single-nucleotide polymorphism; QTL, quantitative trait locus.

Bold value indicates "Total" values are the sum of the values for each respective column.

### 3.4 QTL analysis

Correlating the genotypic data analyzed from 1,048 polymorphic SNP markers with the phenotypic data on the basis of lesion length as well as BB score for the selected 94 RILs, we have identified the major locus on the long arm of chromosome 11 to be associated with BB resistance reaction. Inclusive composite interval mapping for additive QTL through the IciMapping software marks the presence of QTL between the two SNP markers, Chr11.27817978 and Chr11.27994133, with a LOD score of 10.21 explaining 35.93% of phenotypic variations (**Table 3**). The markers were located at 415 cM on the linkage map and span a physical distance of 176 kb referred on the Nipponbare reference genome IRGSP v1 (**Figure 2**) (**Supplementary Figure 1**).

**Table 3:** Detail about the putative QTL associated with bacterial blight resistance.

Analysis	QTL	Chr	Position (cM)	L Marker	R Marker	LOD	R^2^ (%)	Add
ICIM	qBB_11	11	415 cM	27817978	27994133	10.21	35.92	1.9

ICIM, inclusive composite interval mapping; L Marker, left marker; R Marker, right marker; LOD, l ogarithm of the odds/log_10_ of the ratio of the probability that a QTL is present to the probability that a QTL is absent; R%, percentage of phenotypic variation explained by the QTL; Add, additive effect; QTL, quantitative trait locus.

### 3.5 Identification of candidate genes and their expression analysis

**Figure 3:**
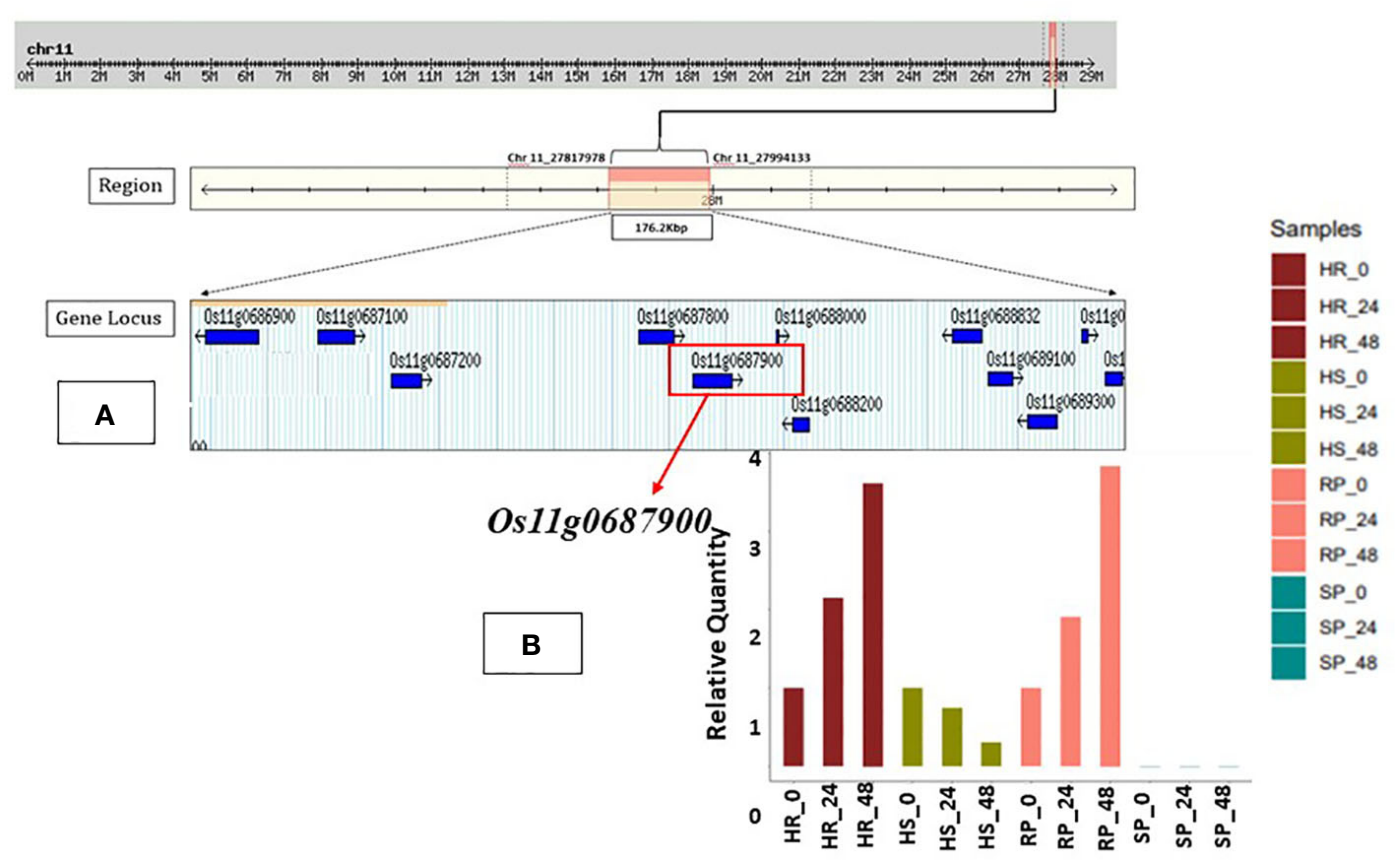
**(A)** *In silico* analysis for the identified QTL associated with resistance against bacterial blight through Rice Genome Annotation Project Database (RAP-DB). Ten putative candidate genes encoding various biological functions were present in the region of major QTL. Source RAP-DB (IRGSP-1.0). **(B)** Fold change and expression level of putative candidate gene *Osllg0687900*. Gene *Osllg0687900* encoding for NB-ARC domain-containing protein; expression level shows upregulation at 24 hpi and 48 hpi for both resistant parent (RP) and homozygous resistant (HR) lines and possibly associated with resistance against bacterial blight. SNP, single-nucleotide polymorphism; QTL, quantitative trait locus.

We analyzed the QTL region of interest for the presence of genes based on the Rice Genome Annotation Project Database and resource. The region indicated the presence of 10 genes encoding various biological functions, which includes two NBS-LRR class disease resistance proteins, two NB- ARC-containing protein, one von Willebrand factor-containing protein, one MLA10 protein, and four hypothetical conserved proteins. Among them, two of the genes are associated with blast disease resistance. The details of the putative candidate genes are described in **Supplementary Table 1**; **Figure 3A**.

The expression analysis of 10 genes from the QTL region associated with BB resistance was investigated through the relative quantification method (also known as the 2^−ΔΔCt^ method) in the parents as well as a highly resistant RIL (RIL-1) and a highly susceptible RIL (RIL-94) line. Notably, the putative candidate gene *Os11g0687900* showed a substantially upregulated expression level (two-fold) in RP and HR in an exponential pattern after inoculation with the *Xoo* isolate IX-020 at 24 hpi and 48 hpi (**Figure 3B**). The expression of other potential genes was either low or absent at other times, depending on the circumstances. The expression details of other candidate genes are also provided in **Supplementary Table 3**. The expression level of gene *Os11g0687900* as well as functional annotation correlated with the identified resistance QTL and was hence considered to be the most probable candidate gene.

### 3.6 Analysis of sequences of the candidate gene *Os11g0687900*

**Figure 4:**
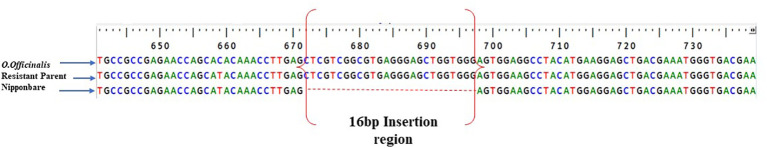
The alignment result depicts the presence of 16-bp insertion region in *Oryza officinalis* genome as well as resistant parent, while Samba Mahsuri revealed the absence of 16-bp insertion and also sequence variation in the region flanking the gene.

The putative candidate gene *Os11g0687900* was sequenced in the resistant parent, IR 75084-15-3-B-B, and susceptible parent Samba Mahsuri using a set of seven designed overlapping primers. The sequence analysis revealed that there was 16-bp insertion in the 5′UTR of resistant parent IR 75084-15-3-B-B with respect to the Nipponbare sequence. The insertion is located 23 bp upstream of the start codon of the gene, within the 5′UTR. Notably, PCR amplification using the overlapping primers did not yield any product with Samba Mahsuri. Further, a comparative analysis of the genome of Samba Mahsuri (unpublished and Reddy and Ulaganathan, 2015), Nipponbare, and the resistant parent revealed that the locus of the gene and its upstream region is absent in Samba Mahsuri. The sequence alignment with the *O. officinalis* genome available in the National Center for Biotechnology Information (NCBI) database (*Oryza_officinalis_v1.0* GCA_008326285.1) showed the presence of the 16-bp insertion in the *O. officinalis* allele of *Os11g0687900*. The alignment result is depicted in **Figure 4**.

Population genomics analysis using the tool RiceVarMap (Zhao et al., 2021) indicated that approximately 42.74% of the 4,726 sequenced rice varieties do not possess this locus, and approximately 47.20% of the varieties do not possess the 16-bp insertion. Additionally, the population analysis indicated that insertion at this locus is sparsely distributed (~8.6%) in the rice germplasm.

Considering the following observations in the present study (viz., i) gene expression of *Os11g0687900* was exponentially upregulated upon *Xoo* infection and ii) a 16-bp insertion is present in the 5′UTR of the gene only in the BB-resistant donor parent), it is indeed compelling to speculate that this insertion may have a regulatory role with respect to the induction of the gene observed only in resistant plants. The 16-bp insertion sequence was predicted to harbor AP2/ERF transcription factor binding site using the PLACE database (New PLACE —A Database of Plant Cis-acting Regulatory DNA Elements) (Higo et al., 1999), which is frequently used for the identification of plant *cis*-acting regulatory DNA elements.

### 3.7 Development of functional marker for *Xa48t*

**Figure 5:**
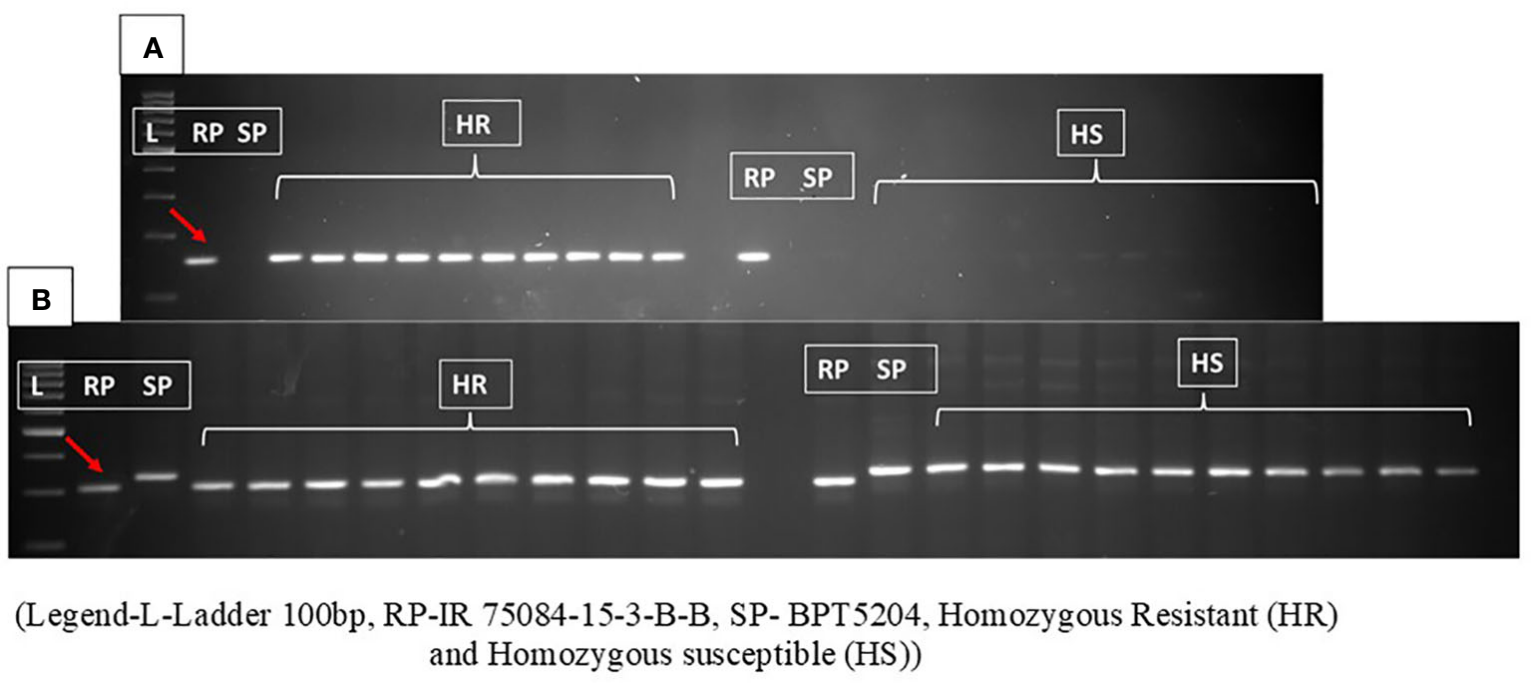
**(A)** The amplification pattern of primer designed 16-bp insertion shows band size of approximately 150 bp representing the resistance in parent and homozygous resistant RILs, while PCR was absent in susceptible parent and homozygous susceptible RILs. **(B)** Amplification pattern of functional Marker_*Xa48* in resistant parent, susceptible parent, homozygous resistant (HR), and homozygous susceptible (HS) RILs. The polymorphic INDEL primers amplify a fragment of size of 316 bp in the susceptible parent and all the susceptible RILs and a 300-bp fragment in the resistant parent and the resistant RILs with complete co­segregation in the mapping population. RILs, recombinant inbred lines. Red arrow represents the band size of resistant parent.

The functional markers for the mapped gene were developed and validated in the population. In the first set, primers were designed from the 16-bp insertion region that clearly showed 150-bp amplification in the resistant parent and homozygous resistant RILs, while the PCR amplicon was absent in the susceptible parent and homozygous susceptible RILs. These results further indicated that the 16-bp insertion-associated region was present only in the RP and HR and absent in the SP and HS (**Figure 5A**).

In order to develop co-dominant markers for marker-assisted breeding, five SSR markers and eight INDEL markers in the vicinity of the candidate gene for *Xa48t*, viz., *Os11g0687900*, were used. One of the INDEL markers was found to be polymorphic between the resistant parent and the susceptible parent. The polymorphic INDEL marker was located at a distance of approximately 78 kb from locus *Os11g0687900*. The polymorphic INDEL primers amplify the band size of 310 bp in the susceptible parent and 300 bp in the resistant parent and also showed complete co-segregation with the phenotype in the RIL mapping population (**Figure 5B**). The INDEL marker is named Marker_*Xa48* and can be used as a functional marker for gene *Xa48t*.

## 4 Discussion

Deployment of a diverse set of resistance genes represents the most feasible strategy to ensure durable management of BB disease of rice. In previous studies, at least 47 resistance genes have been mapped from diverse sources. Few major resistance genes have been mapped from wild species of *Oryza* and also characterized. These include *O. longistaminata*; *Xa21* (Song et al., 1995), *O. minuta*; *Xa27*, *Xa35(t)* (Gu et al., 2004; Guo et al., 2010), *O. rufipogon*; *Xa30* (Tan et al., 2004), *O. officinalis*; *Xa29(t)* (Tan et al., 2004), *O. nivara*; *Xa33* (Natrajkumar et al., 2012), *O. nivara*; *Xa38*, *Oryza australiensis*; and *Xa40(t)* (Kim et al., 2015). However, because of the breakdown of resistance due to the existence/emergence of virulent races of *Xoo*, the breeding of rice cultivars that have durable resistance is a challenge (Busungu et al., 2016; Neelam et al., 2019; Chen et al., 2020). In this regard, expanding the repertoire of resistance genes through tapping the wild rice gene resources and pyramiding two or more resistance genes to develop BB resistance cultivars represent the ideal strategy for the management of the disease (Sundaram et al., 2009). In this study, we have identified and mapped the novel gene locus conferring resistance against bacterial blight disease on the long arm of chromosome 11 from the rice breeding line having introgression segments from *O. officinalis*.

Previously, we screened a set of wild rice introgression using different hypervirulent isolates of *Xoo* to identify the potential donor for the development of BB-resistant cultivars. The rice introgression line, IR-75084-15-3-B-B, possessing introgressions from *O. officinalis*, displayed broad-spectrum resistance against the four hypervirulent isolates of the pathogen, namely, IX-020, IX-007, IX-212, and IX-206, under controlled (glasshouse) conditions, indicating broad-spectrum resistance against the disease (Sinha et al., 2021). In order to understand the inheritance of resistance in the rice line and also to undertake molecular mapping of the resistant locus/loci, we developed a mapping population by crossing the resistant rice line IR-75084-15-3-B-B and Samba Mahsuri, which is susceptible to bacterial blight. Based on the segregation pattern of resistance trait in the F_2_ generation, it was inferred that the population is segregating in the ratio of 3: 1, and the resistance is governed by a single, dominant gene.

The RIL mapping population derived from the cross was subsequently phenotypically evaluated against *Xoo*. A subset of 94 RILs in F_7_ generation displaying extreme phenotype reaction (i.e., 47 RILs that are homozygous resistant and 47 RILs that are homozygous susceptible) was selected for high-throughput SNP-based genotyping using 7k rice SNP chip (Morales et al., 2020). The screening of 20 homozygous resistant RILs with four hypervirulent *Xoo* strains showed a similar resistance pattern as that of the resistant parent, suggesting that all the resistant RILs carried the genetic loci for broad-spectrum resistance. Recombinant inbred lines are highly suited for molecular mapping of genes/QTLs, as they do not show any segregation of individuals within RILs (Rockman and Kruglyak, 2008). The high-density linkage mapping and the gene/QTL analysis based on the high-throughput SNP genotyping of 94 RILs laid a strong foundation in our study for the identification of genomic regions associated with bacterial blight resistance trait. This approach has been adopted in earlier studies aimed at the molecular mapping of genes/QTLs associated with agronomically important genes/QTLs in rice (Kim, 2018; Kim and Reinke, 2019; Neelam et al., 2019).

From the correlation analysis of phenotypic and genotypic data of 94 RILs, we identified and mapped a novel genetic locus that governs resistance against bacterial blight on the long arm of chromosome 11 (https://www.isbreeding.net). The resistance locus, which is named *Xa48*, was flanked by SNP markers 11955796 and 11963686 spanning a distance of 176 kb (i.e., from 27.81 Mbp to 27.99 Mbp) on chromosome 11. So far, about 17 of the previously identified bacterial blight resistance genes including *Xa3*/*Xa26*, *Xa4*, *Xa10*, *Xa21*, *Xa22*, *Xa23*, *xa26*, *Xa30*, *Xa32*, *Xa35*, *Xa36*, *Xa39*, *Xa40*, *Xa43(t)*, *xa44(t)*, and *Xa47(t)* were also localized on chromosome 11 (Yoshimura et al., 1983; Song et al., 1995; Kaku and Ogawa, 2001; Wang et al., 2001; Zhang et al., 2001; Chen et al., 2002; Wang et al., 2003; Sun et al., 2004; Tan et al., 2004; Guo et al., 2010; Miao et al., 2010; Zhang et al., 2014; Kim et al., 2015; Kim, 2018; Kim and Reinke, 2019 and Xing et al., 2021). Among these genes, the gene locus identified in this study was found to be located in the vicinity of five genes, namely, *Xa3*/*Xa26*, *Xa4*, *Xa40*, *Xa43(t)*, and *Xa47(t)* (Wang et al., 2001; Sun et al., 2004; Kim et al., 2015; Kim and Reinke, 2019). Among them, the earlier identified genes, *Xa3*/*Xa26*, *Xa4*, and *Xa40*, have been mapped and characterized in a region not very close to the gene identified in this study, i.e., *Xa48*, while *Xa43(t)* is located in the proximity of *Xa48* in the upstream region, and *Xa47(t)* is located in the downstream region. However, the PCR-based diagnostic markers developed for *Xa43(t)*, i.e., IBb27os11_14 and S_BB11.ssr_9, did not show polymorphism among the parents used in this present study (Kim and Reinke, 2019). Also, while *Xa43(t)* was identified from the rice line, P8 using a Japonica MAGIC population (Bandillo et al., 2013), the gene identified in this study, i.e., *Xa48*, has been identified from a wild species of rice, *O. officinalis*. Another newly identified gene locus *Xa47(t)* was identified from introgression line G252 from wild rice *O. rufipogon* against virulent strains of China, Japan, and the Philippines. A lso, the identified candidate gene for *Xa47(t)* gene is *Os11g0688832*, which is different from *Xa48* putative candidate gene. This indicates that *Xa48* is indeed a novel BB resistance gene.

The information obtained through the Rice gene annotation project database (Sakai et al., 2013) is useful for identifying putative candidate genes located in the genomic region spanning genes/QTLs (Kale et al., 2021). Through *in silico* analysis, a total of 10 putative candidate genes encoding different biological functions were found to be located in the region of interest. The functional annotation indicates the presence of a distinct class of genes encoding proteins with various biological functions in the 176 kb region. The NBS-LRR domain plays an important role in signal transduction and mediates effector-triggered immunity against a diverse array of pathogens (Hammond Kosack and Jones 1996). Another gene encoding for the NB-ARC domain is also involved in activating defense response by acting as signaling motifs (van Ooijen et al., 2008). The gene encoding for MLA10 functions in regulating cell compartment-specific activity in cell death and disease resistance in barley (Bai et al., 2012). Other genes were involved in the molecular and structural activity via protein binding. These genes are worth investigating further through expression analysis to establish their putative role in bacterial blight resistance.

Among the genes underlying mapped QTL, expression analysis was carried out to identify the promising candidate gene(s) associated with the resistance phenotype. The results based on the relative quantification of genes in different samples revealed that the expression of *Os11g0687900* was significantly higher in the resistant parent IR-75084-15-3-B-B and homozygous resistant lines as compared to the bacterial blight- susceptible parent Samba Mahsuri and homozygous susceptible line. The functional annotation of this gene suggests an NB-ARC domain-containing protein, which is known to be associated with disease resistance (van Ooijen et al., 2008). Further investigation through sequencing of the resistance and susceptible alleles of *Os11g0687900* among the resistant and susceptible parents revealed the insertion of 16 bp in the 5′UTR of the resistant parent. The 16-bp insertion is unique to the resistant parent and also identified in the *O. officinalis* genome, suggesting that this *cis*-acting motif of 16 bp might play a critical role in bacterial blight resistance. Notably, this motif is predicted to harbor transcription factor binding sites/*cis*-elements, which might be associated with the induction of expression of the gene upon *Xoo* infection (Jiang et al., 2020). Further, analysis of the 16-bp motif in the publically available genome sequence of *O. officinalis*, along with Nipponbare and several Indica rice varieties, indicated the absence of 16-bp insertion region in the majority of Indica rice cultivars including Samba Mahsuri. It would be interesting to further validate the precise function of *Os11g0687900* in BB resistance by over-expression or knockout through genome editing. In particular, the *cis*-acting motif of 16 bp is an interesting subject for further investigation. Knock-in (using homology-directed repair (HDR) of genome editing) of the 16-bp motif in popular and susceptible rice cultivars can help develop resistant alleles in susceptible cultivars. A PCR-based INDEL marker, named Marker_*Xa48*, which is located at a distance of 78 kb from the gene locus, has been developed and shown to be co-segregating with the resistant phenotype. This marker can be used for introgression of resistant alleles of the *Xa48* gene through MAB.

## 5 Conclusion

Based on the findings in our study, the major locus on the long arm of chromosome 11 associated with BB resistance reaction has been identified from *O. officinalis* introgression lines through 7k SNP chip-based ICIM mapping and was named *Xa48t*. The resistance locus was observed to be located between two SNP markers, Chr11.27817978 and Chr11. 27994133, explaining 35.93% of phenotypic variation and with a LOD score of 10.21. The most promising candidate gene associated with the resistant trait is *Os11g0687900*, which codes for the NB-ARC domain-containing protein, and its candidacy has been validated through expression analysis. The PCR-based INDEL marker tightly linked to gene *Xa48t* has been developed for the deployment of the gene into elite rice cultivars through marker-assisted breeding.

## Data availability statement

The datasets presented in this study can be found in online repositories. The names of the repository/repositories and accession number(s) can be found below: https://www.ncbi.nlm.nih.gov; OQ559125 (BankIt2678609 Oryza; OQ559125).

## Author contributions

PS carried out disease screening, mapping, expression analysis, and functional marker development and drafted the manuscript. DKT carried out disease screening and crossing program and drafted the manuscript. MS interpreted the expression analysis data. CG assisted with analysis and functional marker development. HS, AD, AM, RG, PE, KM, MS, CK, and YA helped with disease screening in the field. SKM guided the mapping and expression analysis and provided critical input in the manuscript. LG and CN guided the mapping and expression analysis and reviewed the manuscript. HP, IG, S-RK, KJ, SRH, and RO revised the manuscript. SRM conceptualized the experiment; guided disease screening, mapping, expression analysis, and functional marker development; and critically revised the manuscript. All authors contributed to the article and approved the submitted version.
