# Supplementary material for: Fine mapping and sequence analysis reveal a promising candidate gene encoding a novel NB-ARC domain derived from wild rice (Oryza officinalis) that confers bacterial blight resistance
Source: Front Plant Sci. 2023 Aug 25;14:1173063. doi: 10.3389/fpls.2023.1173063 (PMC10485833; doi:10.3389/fpls.2023.1173063)
Supplement: Supplementary file 1 [file DataSheet_1.docx]

Supplementary Material

Fine mapping and sequence analysis reveals a promising candidate gene encoding a novel NB-ARC domain derived from wild rice (*Oryza officinalis*) confers bacterial blight resistance

Pragya Sinha^1^ ^†^, Dilip Kumar T^1 †^, Hajira Sk^1^, Manish Solanki^1^, CG Gokulan^2^, Ayyappa D^1^, Anila M^1^, Rekha G^3^, Punniakoti E^1^, Kousik MBVN^1^, Masthani SK^1^, Chaitra K^1^, Yugander A^1,10^, Laha GS^1^, Chirravuri N Neerja^1^, Hitendra Kumar Patel^4^, Irfan Ahmad Ghazi^5^, Sung-Ryul Kim^6^, Kshirod K. Jena^7^, H Surekha Rani^8^, Ricardo Oliva^9^, Satendra K Mangrauthia^1^*, and Sundaram RM^1*^.

^1^ ICAR-Indian Institute of Rice Research, Rajendranagar, Hyderabad, India.

^2^ CSIR-Centre for Cellular and Molecular Biology, Hyderabad, India.

^3^ Rallis India Limited, Seeds/Biotech R&D Division, Bangalore, India.

^4^ Academy of Scientific and Innovative Research (AcSIR), Ghaziabad, India

^5^ Department of Plant Sciences, School of Life Sciences, University of Hyderabad, Hyderabad, India

^6^ Rice Breeding Innovation Platform, International Rice Research Institute (IRRI), Los Banos, Philippines

^7^ School of Biotechnology, KIIT University, Bhubaneswar 751024, Odisha, India

^8^ Department of genetics, Osmania University

^9^ Safe and Sustainable Value Chain, World Vegetable Center

^10^ Institute of Molecular Physiology, Heinrich Heine University, Universitätsstraße 1, 40225 Düsseldorf, Germany

*** Correspondence:**Dr. R M Sundaram and Dr. Satendra K Mangrauthia.
rms_28@rediffmail.com, skmdrr@gmail.com.

† Equally Contributed.

# Table 1: List of RT-Primer for expression analysis

| Locus | **Function** | Forward Primer | Reverse Primer | Amplicon size | Annealing temp |
| --- | --- | --- | --- | --- | --- |
| Os11g0686900 | NBS-LRR disease resistance protein, putative, expressed | AAGGGCTGAAGAGTTGGCGTTC | GCCTCACGCATTGTAAAGTGATGC | 80 | 61.6 |
| Os11g0687100 | protein binding protein | AGACTCGGTTGGACGATTTGCAG | AGCTACACTCATCGCCTCTGTG | 118 | 63.1 |
| Os11g0687200 | von Willebrand factor type A domain containing protein | TGGCGGCGTACATATTCTCGTG | AACCTCCAGCGTCACGAACAAG | 104 | 66.3 |
| Os11g0687800 | MLA10, putative, expressed | CATTCTACTGCCGTTGTTCTCTGC | ATTTGCCCGTCTGCTCCACAAC | 123 | 61.6 |
| Os11g0687900 | NB-ARC domain containing protein | AGCTGAGGACTTGCTCTCTGTACC | TGTCAGCCGCTTCCTGCTAATC | 71 | 60.6 |
| Os11g0688000 | conserved hypothetical protein | TCGGAGAATCGACTGCAGAAGG | TGAGCTTGTAGATGGCGAGCTTTC | 72 | 60.6 |
| Os11g0688200 | retrotransposon protein | TTAACCCACCTCAAGGCGAAGACC | TTCGCCGTGTATCACGCCTATG | 69 | 60.4 |
| Os11g0688832 | Leucine Rich Repeat family protein | ACCATGGCATGATAAGGAGCAAAG | ACTGTTCCGATTGTCCGGGAAG | 79 | 61.9 |
| Os11g0689100 | NB-ARC domain containing protein, | AGCCACCAGTTGATGTGTCTGC | CAGCTGAGAGTTTCCTTGGCCTTG | 69 | 61.8 |
| Os11g0689300 | calmodulin-binding protein MPCBP | GCAACAATGCATGCAGAAGGTTAC | CATTGACGTAGGCAGCCAATGC | 76 | 61.6 |

# Table 2: List of Primers designed and used for the marker development.

| Primer | Forward Sequencce | Reverse Sequence | Remarks | Result |
| --- | --- | --- | --- | --- |
| Primer | GAGAACAAATCCGGTCCAGC | CGTCTTGGTATGTCTCGTTCG | Dominant Primer | Dominant amplification with resistant genotype |
| RM27340 | GTGATGCTGTGCCGATAATATTCC | AGCAGTGATTCATCGCTCTATCG | SSR | Monomorphic |
| RM27342 | TCATCTTCATCGGTGTCTTCC | CTGAAGGAAGTTCAAGAGGATGG | SSR | Monomorphic |
| RM27348 | GGAGGCAATGGTAGAACTTCAGG | GGACGGTAGGAGATCCATGTCG | SSR | Monomorphic |
| RM27352 | GGAGGCAATGGTAGAACTTCAGG | GGACGGTAGGAGATCCATGTCG | SSR | Monomorphic |
| RM27353 | ACGCAGCAGCATTTCAAGACG | TAACAGGACCCAGTGGAATCTGG | SSR | Monomorphic |
| Indel Marker_1 | TGATCGGAACATCTGGACACA | AGGATCTTGATGTTGCCTAATGG | INDEL | Not Amplified |
| Indel Marker_2 | TGGGCCTCACATGTCAGT | GAGAGGTTTCGCTGATGTGG | INDEL | Polymorphic |
| Indel Marker_3 | ACCACCTTGAGCTTCTCCTC | CGACAAAGTCCGTGGTATCC | INDEL | Not Amplified |
| Indel Marker_4 | CATTTGATTGGGAGGGGTGC | CAATACCTCTTTGATGCATGCA | INDEL | Not Amplified |
| Indel Marker_5 | GCCATGCTCTGAAGATTCCA | TGCGATCAGTTTGAGGTAAAGA | INDEL | Monomorphic |
| Indel Marker_6 | GGGCTAGCTCTTCCACTCAG | CGAACGAAGACAAGCGAGA | INDEL | Not Amplified |
| Indel Marker_7 | GAGAGGAGAAGCGGGTTGTA | TGAGCCTCGTCGTATATGGT | INDEL | Monomorphic |
| Indel Marker_8 | GGAGGAGTCGATGCTGGAG | GTCTCGTGCACCATCTCCT | INDEL | Monomorphic |

# Table 3: Relative Quantity of Samples with genes recorded at different time intervals.

| Samples | HR | | | HS | | | RP | | | SP | | |
| --- | --- | --- | --- | --- | --- | --- | --- | --- | --- | --- | --- | --- |
| Time interval (in hpi) | 0 | 24 | 48 | 0 | 24 | 48 | 0 | 24 | 48 | 0 | 24 | 48 |
| Gene |  |  |  |  |  |  |  |  |  |  |  |  |
| Os11g0686900 | 1 | 7.58 | NA | NA | NA | NA | 1 | 7.91 | NA | 1 | NA | 3.64 |
| Os11g0687900 | 1.00 | 2.15 | 3.62 | 1.00 | 0.74 | 0.30 | 1.00 | 1.91 | 3.84 | 0.00 | 0.00 | 0.00 |
| Os11g0687800 | 1.00 | 6.38 | 6.53 | 1.00 | 1.00 | 2.14 | 1.00 | 0.97 | 2.14 | 1.00 | 1.00 | 2.46 |
| Os11g0687200 | 1.00 | 6.64 | 9.85 | 1.00 | 0.74 | 0.61 | 1.00 | 0.81 | 1.00 | NA | NA | NA |
| Os11g0688000 | 1.00 | 7.63 | NA | NA | NA | NA | 1.00 | 1.57 | 0.01 | NA | NA | NA |
| Os11g0689300 | 1.00 | 0.87 | 1.00 | 1.00 | 0.51 | NA | NA | NA | NA | 1.00 | 0.69 | 89.66 |

# Legend HR- Homozygous resistant, HS- Homozygous susceptible, RP- Resistant Parent, SP-Susceptible Parent, hpi-hours post-inoculation.

**Supplementary Figure 1: LOD Profile of major QTL on chromosome 11 associated with BB resistance analysed by ICIM.**

**
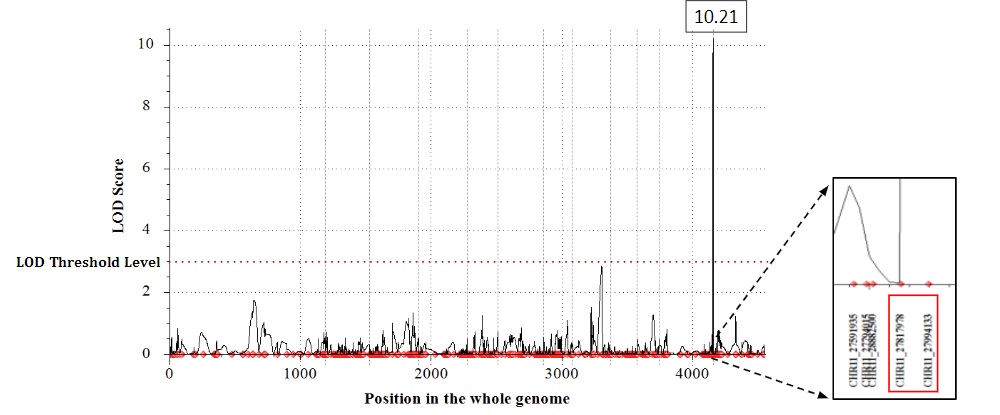
**

For the identified QTL on chromosome 11 between the SNP markers Chr11_27817978 (left marker) and Chr11_27994133 (right marker) have shown LOD score of 10.21.

**Supplementary table 4: Reaction of selected RILs to multiple hypervirulent *Xoo* isolates.**

| **RILs** | **IX-020** | **IX-007** | **IX-212** | **IX-206** |
| --- | --- | --- | --- | --- |
| IR 75084-15-3-B-B (RP) | 1.5±0.9 (1) | 2.08±0.9 (1) | 1.12±0.3 (1) | 1.96±0.7 (1) |
| SM(SP) | 6.8±0.9 (7) | 7.44±0.3 (7) | 9.9±0.6 (9) | 8.84±0.7 (7) |
| RIL-1 | 1.72±0.34 (1) | 1.85±0.8 (1) | 0.89±0.2 (1) | 1.29±0.4 (1) |
| RIL-2 | 1.56±0.39 (1) | 2.25±0.7 (1) | 1.41±0.4 (1) | 2.41±1.1 (1) |
| RIL-3 | 1.83±0.36 (1) | 2.02±0.6 (1) | 1.01±0.2 (1) | 1.82±0.3 (1) |
| RIL-4 | 2.14±0.27 (1) | 1.98±0.9 (1) | 1.31±0.3 (1) | 1.64±0.9 (1) |
| RIL-5 | 1.61±0.38 (1) | 2.15±0.8 (1) | 0.98±0.4 (1) | 2.01±0.6 (1) |
| RIL-6 | 1.49±0.42 (1) | 2.01±0.9 (1) | 1.24±0.3 (1) | 1.54±1.4 (1) |
| RIL-7 | 1.75±0.33 (1) | 1.92±0.7 (1) | 0.95±0.2 (1) | 2.25±0.9 (1) |
| RIL-8 | 1.97±0.31 (1) | 2.10±0.6 (1) | 1.38±0.4 (1) | 1.19±0.8 (1) |
| RIL-9 | 1.63±0.37 (1) | 2.05±0.8 (1) | 1.08±0.2 (1) | 2.02±0.5 (1) |
| RIL-10 | 1.89±0.35 (1) | 2.12±0.9 (1) | 1.28±0.3 (1) | 1.71±0.7 (1) |
| RIL-11 | 1.78±0.32 (1) | 1.93±0.7 (1) | 0.92±0.4 (1) | 1.93±1.3 (1) |
| RIL-12 | 1.55±0.41 (1) | 1.96±0.6 (1) | 1.35±0.3 (1) | 1.74±0.4 (1) |
| RIL-13 | 2.02±0.30 (1) | 2.19±0.9 (1) | 0.82±0.2 (1) | 2.12±1.2 (1) |
| RIL-14 | 1.67±0.36 (1) | 2.07±0.8 (1) | 1.51±0.4 (1) | 1.47±0.6 (1) |
| RIL-15 | 1.91±0.34 (1) | 1.91±0.9 (1) | 1.05±0.2 (1) | 2.06±0.8 (1) |
| RIL-16 | 1.79±0.31 (1) | 2.28±0.7 (1) | 1.45±0.3 (1) | 1.88±0.5 (1) |
| RIL-17 | 1.58±0.40 (1) | 1.99±0.6 (1) | 1.11±0.4 (1) | 1.39±1.2 (1) |
| RIL-18 | 2.09±0.28 (1) | 2.30±0.9 (1) | 1.29±0.3 (1) | 2.17±0.7 (1) |
| RIL-19 | 1.69±0.35 (1) | 1.97±0.8 (1) | 0.93±0.2 (1) | 1.76±0.9 (1) |
| RIL-20 | 1.87±0.33 (1) | 2.23±0.7 (1) | 1.37±0.4 (1) | 2.04±0.4 (1) |
| RIL-21 | 6.5±0.8 (7) | 7.23±0.2 (7) | 9.6±0.5 (9) | 8.24±0.9 (7) |
| RIL-22 | 6.2±0.7 (7) | 7.67±0.4 (7) | 10.3±0.7 (9) | 9.31±0.5 (7) |
| RIL-23 | 6.3±0.6 (7) | 7.36±0.2 (7) | 9.8±0.5 (9) | 8.47±0.6 (7) |
| RIL-24 | 7.2±0.9 (7) | 7.58±0.3 (7) | 10.2±0.6 (9) | 8.97±0.8 (7) |
| RIL-25 | 6.9±0.8 (7) | 7.32±0.4 (7) | 9.7±0.7 (9) | 8.58±0.6 (7) |
| RIL-26 | 6.7±0.9 (7) | 7.52±0.3 (7) | 10.1±0.6 (9) | 9.05±0.7 (7) |
| RIL-27 | 6.4±0.7 (7) | 7.29±0.2 (7) | 9.4±0.5 (9) | 8.39±0.8 (7) |
| RIL-28 | 7.0±0.6 (7) | 7.63±0.4 (7) | 10.5±0.7 (9) | 9.11±0.6 (7) |
| RIL-29 | 6.6±0.8 (7) | 7.40±0.2 (7) | 9.7±0.5 (9) | 8.73±0.9 (7) |
| RIL-30 | 7.1±0.9 (7) | 7.55±0.3 (7) | 10.3±0.6 (9) | 8.92±0.6 (7) |
| RIL-31 | 6.1±0.7 (7) | 7.26±0.4 (7) | 9.5±0.7 (9) | 8.31±0.7 (7) |
| RIL-32 | 6.0±0.6 (7) | 7.49±0.3 (7) | 10.4±0.6 (9) | 9.18±0.5 (7) |
| RIL-33 | 7.3±0.9 (7) | 7.17±0.2 (7) | 9.2±0.5 (9) | 8.14±0.9 (7) |
| RIL-34 | 6.8±0.8 (7) | 7.70±0.4 (7) | 10.7±0.7 (9) | 9.40±0.6 (7) |
| RIL-35 | 6.5±0.9 (7) | 7.33±0.2 (7) | 9.6±0.5 (9) | 8.65±0.8 (7) |
| RIL-36 | 7.4±0.7 (7) | 7.61±0.3 (7) | 10.3±0.6 (9) | 9.07±0.6 (7) |
| RIL-37 | 6.7±0.6 (7) | 7.38±0.4 (7) | 9.9±0.7 (9) | 8.52±0.9 (7) |
| RIL-38 | 7.5±0.9 (7) | 7.56±0.3 (7) | 10.2±0.6 (9) | 9.23±0.6 (7) |
| RIL-39 | 6.3±0.8 (7) | 7.25±0.2 (7) | 9.8±0.5 (9) | 8.79±0.7 (7) |
| RIL-40 | 6.6±0.7 (7) | 7.68±0.4 (7) | 10.3±0.7 (9) | 8.95±0.8 (7) |

Under glass house condition, the selected RILs (20 homozygous resistant (RIL-1 to RIL-20) and 20 homozygous susceptible (RIL-21 to RIL 40) were screened using four hypervirulent isolates (IX-020, IX-007, IX-212 and IX-206). The figures in parentheses indicate disease score.
